# The Synthesis and Characterisation of the High-Hardness Magnetic Material Mn_2_N_0.86_

**DOI:** 10.3390/ma15217780

**Published:** 2022-11-04

**Authors:** Shoufeng Zhang, Chao Zhou, Xin Wang, Kuo Bao, Xingbin Zhao, Jinming Zhu, Qiang Tao, Yufei Ge, Zekun Yu, Pinwen Zhu, Wei Zhao, Jia’en Cheng, Teng Ma, Shuailing Ma, Tian Cui

**Affiliations:** 1 State Key Laboratory of Superhard Materials, College of Physics, Jilin University, Changchun 130012, China; 2 Institute of High Pressure Physics, School of Physical Science and Technology, Ningbo University, Ningbo 315211, China

**Keywords:** Mn_2_N_0.86_, high-temperature and high-pressure synthesis, coexistence of antiferromagnetic and ferromagnetism

## Abstract

High-quality *P*6_3_22 Mn_2_N_0.86_ samples were synthesised using a high-pressure metathesis reaction, and the properties of the material were investigated. The measurements revealed that the Vickers hardness was 7.47 GPa, which is less than that predicted by commonly used theoretical models. At low air pressure, Mn_2_N_0.86_ and MnO coexist at 500 to 600 °C, and by excluding air, we succeeded in producing Mn_4_N by heating Mn_2_N_0.86_ in nitrogen atmosphere; we carefully studied this process with thermogravimetry and differential scanning calorimetry (TG-DSC). This gives a hint that to control temperature, air pressure and gas concentration might be an effective way to prepare fine Mn-N-O catalysis. Magnetic measurements indicated that ferromagnetism and antiferromagnetism coexist within Mn_2_N_0.86_ at room temperature and that these magnetic properties are induced by nitrogen vacancies. Ab intio simulation was used to probe the nature of the magnetism in greater detail. The research contributes to the available data and the understanding of Mn_2_N_0.86_ and suggests ways to control the formation of materials based on Mn_2_N_0.86_.

## 1. Introduction

Transition metal nitrides have attracted much attention owing to their excellent electronic and magnetic properties, hardness, and potential applications [1,2,3,4]. Currently, hermetic sintering [5,6], the arc method [7,8,9], and film deposition [10] are commonly used to produce these compounds. However, all of these methods have drawbacks: nitrogen vacancies inevitably arise during hermetic sintering and with the arc method because of the nature of nitrogen bonding and the high temperature required during syntheses. Apart from this, film deposition methods often result in films with properties that differ from those of bulk samples [11]. Therefore, high pressure is an effective way to prevent nitrogen from escaping [12], and high-temperature and high-pressure (HTHP) synthesis is an effective means of preparing fine nitrides [13,14].

Mn is an element with abundant electronic and spin states that can enrich the properties of compounds formed with light elements. Compounds of Mn with N can be classified into four categories: θ (MnN), η (Mn_3_N_2_), ε (Mn_4_N), and ζ (Mn_5_N_2_, Mn_2_N, and Mn_2_N_0.86_) [15]. MnN and Mn_3_N_2_ are antiferromagnetic with Néel temperatures of 650 K [16] and 927 K [17], respectively. Mn_4_N has two inequivalent manganese sites in different positions, which causes ferrimagnetism [18]. Mn_2_N_0.86_ has been studied both experimentally and theoretically [19,20,21,22,23], although conflicting results were obtained. An in-depth understanding of Mn_2_N_0.86_ might help us to understand Mn_2_N_0.86_-based catalytic processes [24] and electronic materials [25]. In some studies of compounds consisting of manganese and other light elements [26], the characteristic antiferromagnetic curve was not observed upon the application of an external magnetic field. A study of fine Mn-N was therefore expected to enrich our knowledge and deepen the understanding of the characteristics of these compounds. 

In this paper, we briefly describe our methods, present data and results, discuss the results to provide the necessary perspective, and state conclusions.

## 2. Experimental and Simulation Details

We prepared single-phase Mn-N compounds by mechanically mixing Mn powder (99.95% purity) and NaN_3_ powder in a 2:1 molar ratio. The thoroughly mixed precursor was placed in a precustomised *h*-BN tube that was wrapped in pyrophyllite. The assembly was inserted into a China-type SPD6×600T apparatus, with a platinum rhodium electrode to monitor the sample temperature. This equipment enabled us to accurately measure the phase-transition temperatures and temperature range of the phases.

We pulverised the products into powder with an agate mortar and acquired the X-ray diffraction (XRD) patterns (SmartLab SE D/teX Ultra250 diffractometer: power 3 kW, target material Cu, λ = 1.5404 Å, range from 20° to 90°, scanning rate for 2°/min; Rigaku Company). In order to more accurately determine the proportion of nitrogen in the sample, we used an oxygen nitrogen hydrogen analyser (ONH836, LECO, St. Joseph, MI USA) to test the content of nitrogen in the sample for subsequent testing and analysis. The Vickers hardness of the re-sintered samples was measured with a microhardness tester (HV-1000ZDT, China). The surface morphology of the samples was examined with field-emission scanning electron microscopy (SEM) and energy-dispersive spectrometry (EDS) (Magellan 400, Nanolab Technologies, Milpitas, CA, USA), and the crystalline nature of the synthesized specimen was examined with transmission electron microscopy (TEM, JEM-2100F). Magnetisation hysteresis loops were recorded at temperatures of 5, 100, 200, and 300 K by sweeping the magnetic field from −50 kOe to 50 kOe. The variation in the magnetization with temperature was measured in the range 5–350 K, with a magnetic field of 1000 Oe using a SQUID magnetometer (MPMS-XL7). To explore the temperature stability and the approximate nitrogen concentration, we subjected the samples to thermogravimetric analysis and differential scanning calorimetry (TG-DSC, Netzsch STA 449F3). The microstructures and electronic structures of our samples were simulated with spin-polarised DFT using the Perdew−Burke−Ernzerhof (PBE) exchange–correlation function within the Cambridge Serial Total Energy Package (CASTEP) code [27]. The virtual crystal approximation (VCA) calculations based on a weighting of the contribution of the pseudopotentials according to the site occupancies were used [28]. This VCA approach could, in principle, be used to study any composition in a solid solution.

The electron exchange and correlation were treated using the PBE formulation of the generalised gradient approximation (GGA) [29]. The cut-off energy of 520 eV for a reciprocal-space grid was chosen for the plane wave. The Brillouin zones were sampled by 6 × 6 × 6 *k*-point meshes according to the Monkhorst–Pack scheme for geometric optimisation, which ensured that the total energies per formula unit were well converged. Experiments show that it is magnetic, so in the subsequent calculation, spin polarization was included.

## 3. Results and Discussion

**Figure 1:**
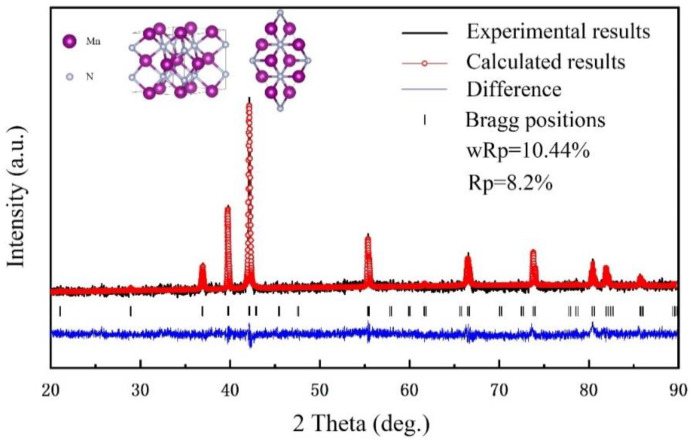
The powder XRD spectrum and the refined structure information of the sample. The black line represents the original experimental data, and the red circles represent the fitting results after Rietveld; ball-and-stick model of the crystal unit cell viewed from different directions.

We prepared initial samples at 5 GPa and 1400 °C with 30 min holding and re-sintered the samples at 500 °C. The re-sintered samples were ground, whereupon their XRD patterns were recorded. The diffraction peaks confirmed that the samples were well crystallised. A comparison of Rietveld’s data [30] with the crystalline data on the standard card indicated that our samples consisted of Mn_2_N_0.86_ in the space group *P*6_3_22, a = 4.8641 (3) Å, c = 4.5269 (2) Å. The XRD pattern and the ball-and-stick structure model are shown in Figure 1. In Mn_2_N_0.86_, each N atom is coordinated with six Mn atoms, and each Mn atom is also coordinated with six N atoms. The crystal structure data are summarised in Table 1. The resistance was recorded throughout the synthesis and re-sintering processes to qualitatively monitor the interaction. At the same time, we found that the sample contained 9.8% N. Considering the range of machine error, we thought that there was a certain nitrogen deficiency in the sample. However, according to the results of refinement, we believed that a small amount of N deficiency would not cause lattice distortion or changes to the crystal space group.

The drastic changes in the resistance at 300 °C and 1200 °C might correspond to the violent outgassing of NaN_3_ and the melting of Mn in the initial raw material. The resistance remained fairly stable at 1400 °C, the optimal temperature at which to synthesise the compound. During the secondary sintering, the resistance increased as the temperature increased to 500 °C, where the resistance reached its most stable point. Although the measurement of the resistance is an elementary approach, it provided an indication of the reactions that were taking place in the chamber.

**Figure 2:**
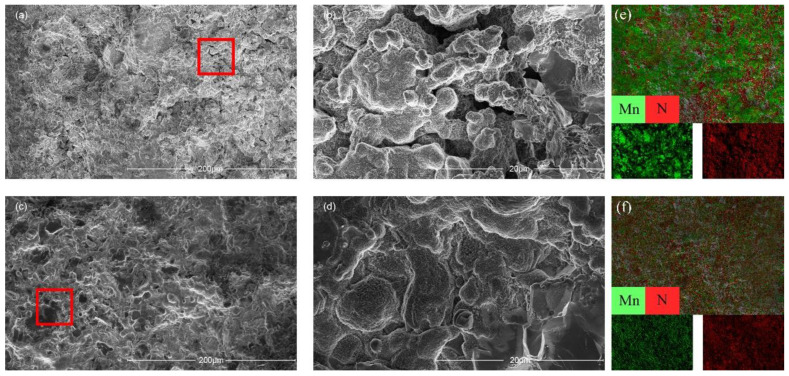
(**a**) A full view of a randomly selected area after the initial synthesis of the sample; (**b**) the enlarged view of the red frame region in (**a**); (**c**) the overall view of another randomly selected region of the sample after secondary sintering and annealing; (**d**) the enlargement of the red frame region of (**c**); (**e**) the element distribution diagram of the initial synthesized EDS of the sample; (**f**) the element distribution diagram of the sample after secondary sintering and annealing by EDS.

**Figure 3:**
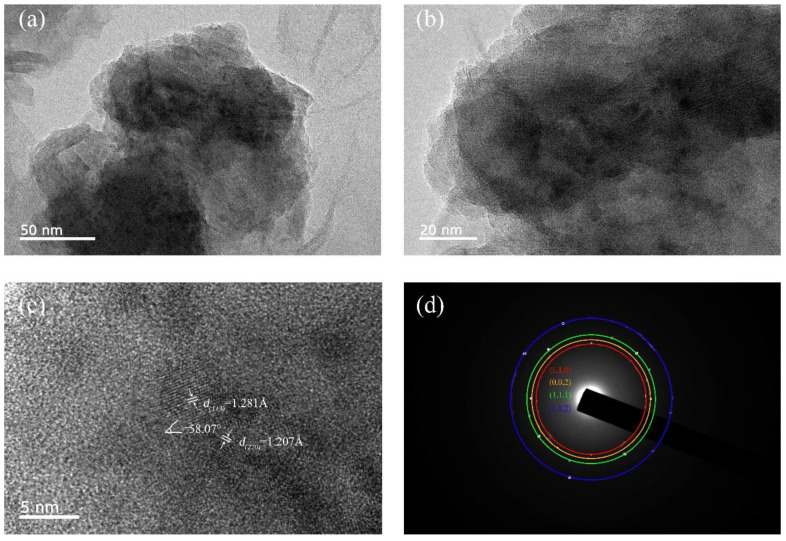
(**a**,**b**) are the bright-field TEM images of the sample. (**c**) The HRTEM of the sample, the planes of (113) and (220), and the angle of two crystal planes. (**d**) The SAED of the sample.

The surface morphology of the sample and the distributions of Mn and N in the samples were examined with SEM and EDS (Figure 2). The quality of the originally synthesised sample was poor in that it was excessively porous and poor crystallinity, which we ascribed to the thermal decomposition of the raw material NaN_3_ to form N_2_. A second sintering process was therefore introduced in an attempt to enhance the quality of the original products. As shown in Figure 2c,d, no obvious pores are visible, and the homogeneity and densification improved. In addition, the EDS analysis of the elemental distribution in the samples after the second sintering indicated that the Mn and N atoms were more evenly distributed, and Na was not detected, as shown in Figure 2e,f. This seemed peculiar considering that NaN_3_ is an important reactant, and the fate of the Na was not immediately clear. Upon closer inspection with the aid of XRD analysis, we found NaO and NaOH in the BN capsules, which means that the BN capsules absorbed the Na atoms from the reactants during the synthesis. This is quite different from other metathesis reactions, such as the formation of CrN [13,14], VN [13], IrN_2_ [31], and OsN_2_ [31]. This difference might be attributed to the N concentration of the product being relatively low and to the fact that the molar ratio of Na is sufficiently low for it to be completely adsorbed by the BN capsules. Therefore, there might be a critical concentration of Na in the metathesis below which the product does not contain any Na. The further characterization of the samples with TEM gave the selected electron area diffraction (SAED) patterns in Figure 3d. The observed circular SAED pattern suggests that the synthesized specimen is of polycrystalline nature. The inter-planar distances calculated from the crystal lattice fringes of high-resolution TEM (HRTEM) shown in Figure 3c are 1.281 Å and 1.207 Å, which correspond to the distances of planes (113) and (220). Meanwhile, the observations of the HRTEM are in line with the measured peaks of the XRD patterns.

**Figure 4:**
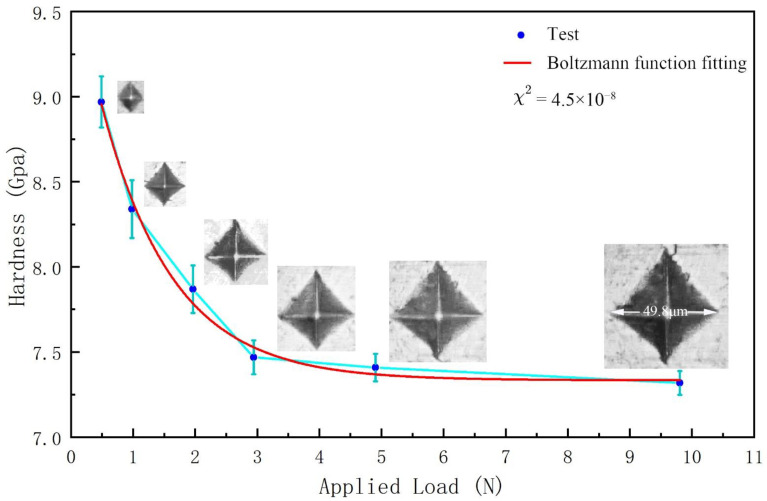
Vickers hardness for Mn_2_N_0.86_. The applied load ranged from 0.49 N (low load) to 9.8 N (high load). Typical optical images of the indentation are shown in the inset.

We measured the Vickers hardness of the re-sintered sample of Mn_2_N_0.86_ and determined its hardness to be 7.47 GPa (Figure 4). As is well known, the hardness is related to the elastic and plastic properties of the material. The relevant mechanical properties were also calculated; for the hexagonal system, the Voigt and Reuss methods were used to evaluate B and G [32]. Several models are available for estimating the hardness of a material [33,34,35,36,37,38], all of which have unique advantages and disadvantages. With Chen’s model [33] of *Hv* = 2(*k*^2^*G*)^0.585^ − 3 (1), *Hv*, *G*, and *B* are the hardness (GPa), shear modulus (GPa), and bulk modulus (GPa), respectively. The parameter *k* is the Pugh’s modulus ratio, namely, *k* = *G/B*; our calculated Vickers hardness of Mn_2_N_0.86_ is 14.26 GPa, which is close to the previously predicted value of 12.01 GPa [20] but significantly larger than the measured value. From Table 2, we can see that by considering magnetism, its bulk elastic modulus is overestimated compared with the previous results [20], which also leads to its B/G > 3. Pugh proposed that the B/G ratio should represent a measure of the machinable behaviour. The critical value that separates ductile and brittle materials is approximately 1.75 [39], and thus, our results revealed that Mn_2_N_0.86_ is still brittle. The predicted Vickers hardness is always higher than the actual hardness for most synthesised transition-metal–light-element compounds [40,41,42,43,44,45,46,47,48,49], regardless of the model that is chosen. We attribute this paradox to the coexistence of high itinerant electrons and the nature of the covalent bonding.

**Figure 5:**
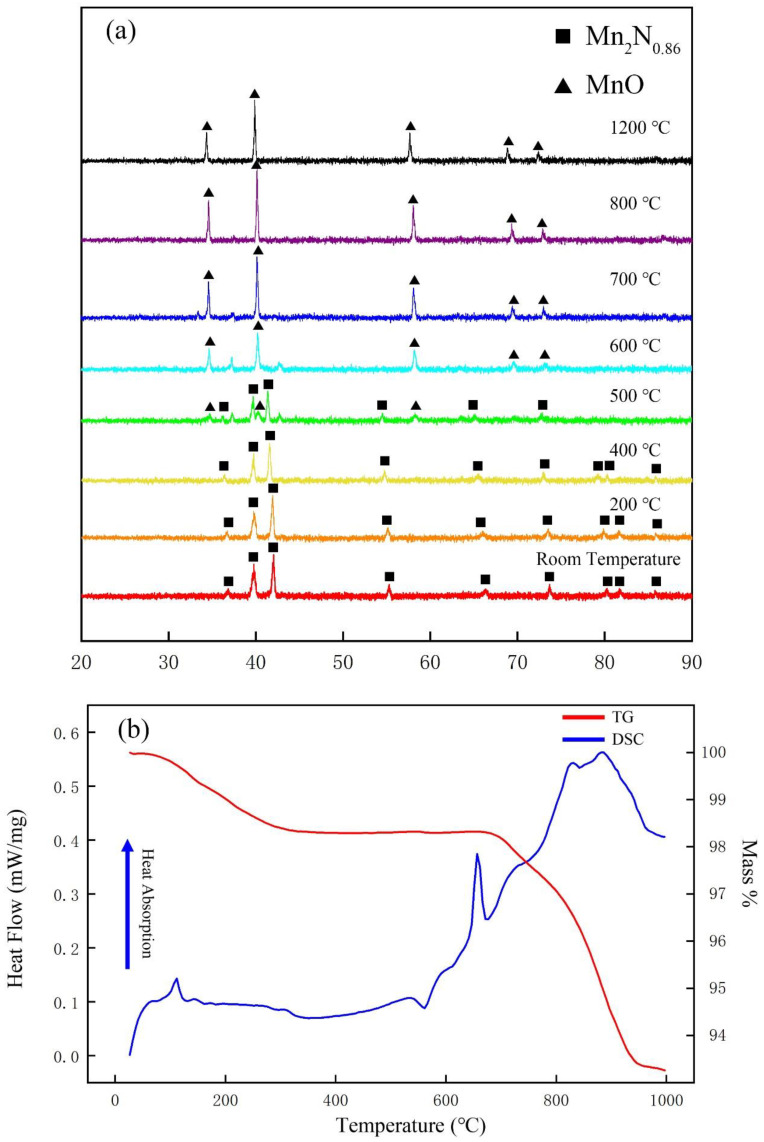
Variable-temperature XRD test data and TG-DSC test data of the samples. (**a**) Variable-temperature XRD test data: The black rectangles mark the Mn_2_N_0.86_ characteristic peaks, and the black triangles mark the MnO characteristic peaks. (**b**) TG-DSC curve of the sample: The red line is the TG curve, and the blue line is the DSC curve.

The sample was analysed by variable temperature XRD with the SE X-ray diffractometer. During this measurement, the sample was mounted on a single crystal low-vacancy Pt sheet, which also functioned as the heating carrier. The results, shown in Figure 5a, indicate that the sample remained stable until 400 °C; at 500 °C, peaks of MnO started appearing; and above 600 °C, only MnO remained. A TG-DSC analysis was conducted from room temperature to 1000 °C at a rate of 10 °C per minute to measure the energy changes the samples underwent as a function of temperature (Figure 5b). The conditions under which this analysis was conducted differed from those of the variable temperature XRD test in that measurements were conducted under a nitrogen gas flow of 30 mL/min. The mass loss of the sample increased during the entire heating process and was greater than 6% overall. Combining the XRD results and the weight of the final residue, we concluded that the final product was Mn_4_N. The TG and DSC curves reveal that obvious weight loss starts at approximately 120 °C; accordingly, an obvious endothermic peak appears on the DSC curve near this temperature. This peak might correspond to the evaporation of the water adsorbed to the sample, which constituted approximately 1.5% of the original mass. The obvious exothermic peak at approximately 550 °C represents an average energy output of approximately 7.795 J/g and might be related to the magnetic phase transition [50]. An intense endothermic peak starts to appear as mass loss commences at 650 °C, which marks the release of nitrogen from the sample. Taking into account the final mass and the XRD results of the residual sample, the heating process from 650 °C to 950 °C was associated with the conversion of Mn_2_N_0.68_ to Mn_4_N. When the heating temperature reached about 900 °C, the N atoms in Mn_4_N began to escape. So far, we have found a method for preparing Mn_4_N. More abundant than previous studies on high-temperature synthesis [51,52], these findings suggest that factors such as the gas pressure of oxygen, gas concentrations of N_2_ and O_2_, and temperature, all of which would be easy to control on an industrial scale, could be varied to control the percentage of MnO-Mn_2_N_0.86_-Mn_4_N to produce the ideal catalytic material MnO-Mn_x_N_y_.

**Figure 6:**
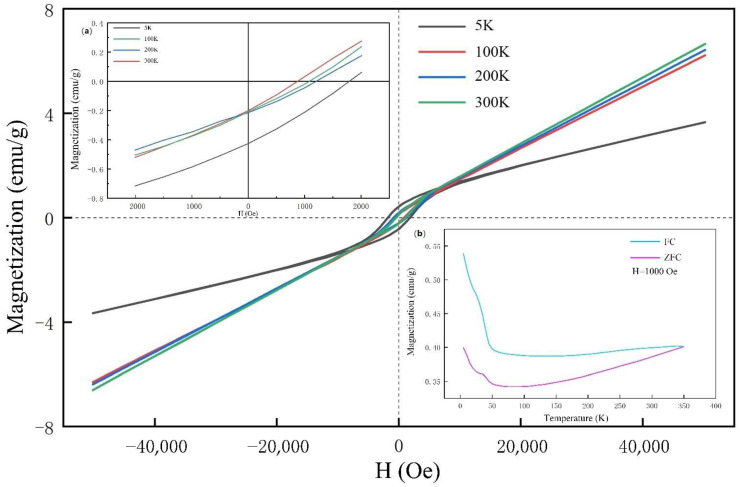
Hysteresis loops for Mn_2_N_0.86_ at 5 K, 100 K, 200 K, and 300 K. (**a**) The M-H curves within the magnetic field of 2000 Oe. (**b**) The temperature dependences of the magnetization for the Mn_2_N_0.86_; the applied magnetic field is 1000 Oe.

We recorded the magnetic hysteresis curves at different temperatures with a field sweep from −50 kOe to 50 kOe (Figure 6, with the inset (b) showing the FC and ZFC curves. The hysteresis decreases with increasing temperature, and the FC curve does not coincide with the ZFC curve. This led us to speculate that ferromagnetism and antiferromagnetism coexisted in the sample simultaneously. This did not correspond with the results from previous studies in which the prepared samples were not single-phased, and the researchers attributed the occurrence of both types of magnetism to impurities [22]. 

**Figure 7:**
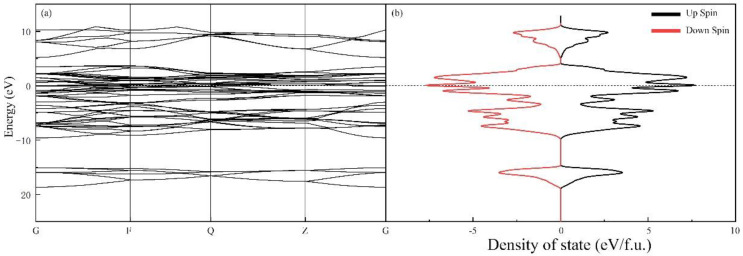
(**a**) Energy band diagram of the sample. (**b**) TDOS diagram of the sample. The Fermi surface has been marked with a dashed line.

To gain a deeper understanding of the co-existence of ferro- and antiferromagnetism in our sample, we conducted first-principle DFT simulations of Mn_2_N_0.86_. The band structure and spin density of states (spin-DOSs), shown in Figure 7, indicate that Mn_2_N_0.86_ is metallic because of the overlapping bands at the Fermi level. Moreover, the DOSs for different spins are symmetric, which means Mn_2_N_0.86_ should be antiferromagnetic. The ferromagnetism might originate from the N vacancies. XRD and EDS are insufficiently sensitive to minute amounts of N vacancies. We simulated the latent heat of Mn_2_N from both the antiferromagnetic and ferromagnetic phases to the nonmagnetic phase with the former being approximately 5% of the latter. Then, using these values to fit the exothermic peak in Figure 5b near 600 K, we determined that the Mn_2_N might be 95% antiferromagnetic and 5% ferromagnetic. Although we do not consider Mn_2_N and Mn_2_N_0.86_ to be equal, the analogy could serve as a helpful indication.

**Figure 8:**
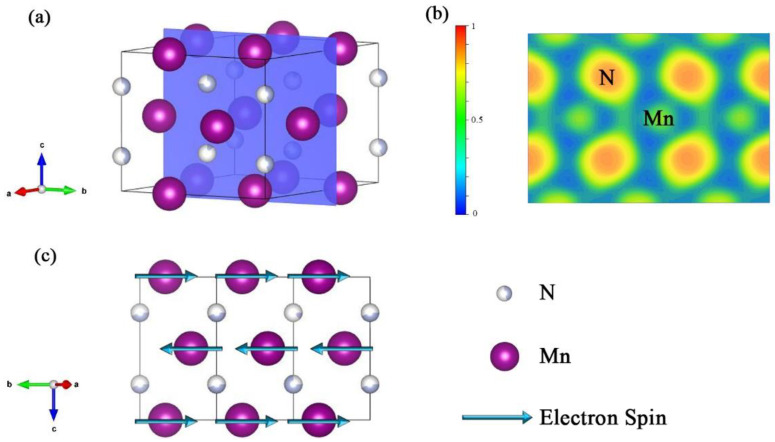
(**a**) The cutting slab of Mn_2_N_0.86_ along the (100) plane. (**b**) ELF of Mn_2_N_0.86_ 2D display for (100) plane. (**c**) Magnetic structure of Mn_2_N_0.86_ projected.

The electronic localisation function (ELF) of the Mn_2_N_0.86_ is shown in Figure 8a,b The electrons between the Mn and N atoms are strongly localised, and the ELF is below 0.5, which corresponds with the value of free electrons. The spin direction of Mn atoms is shown in Figure 8c. As an electron-donating element, Mn transfers most of its electrons to proximate N atoms. This reveals the ionic nature of the Mn-N bond, which is supported by the ELF of Mn_2_N_0.86_, and explains the partially ferromagnetic nature of our sample. Nitrogen vacancies inevitably occur in transition-metal nitrides, and in the case of Mn_2_N_0.86_, N resides at a fractionally occupied position that has the characteristics of a vacancy. The first-principle DFT simulations showed that for Mn_2_N_0.86_, the energy of ferromagnetism is approximately 0.6 eV lower than that of antiferromagnetism. In the case of N vacancies, the system has the tendency to spontaneously evolve to ferromagnetism, which has lower energy. According to the Heisenberg model, an exchange takes place between magnetic atoms, and this energy is expressed as $E_{ex}=-2A\underset{ij}{\sum }S_{i}S_{j}$, where A is the exchange integral, and $S_{i}$, $S_{j}$ is the spin interaction term for the spin at the ith and jth sites. An exchange correlation exists between Mn atoms at the N vacancies in the crystal, where A > 0. Moreover, the spins between Mn atoms that undergo this exchange are parallel, and thus, $S_{i}\cdot S_{j}>0$. This explains the ferromagnetism in the sample.

## 4. Conclusions

We synthesised Mn_2_N_0.86_ samples using high pressure and high temperature. A second sintering process enhanced the quality of the samples, which met the requirements for many types of measurements. The Vickers hardness of the Mn_2_N_0.86_ sample was measured to be 7.47 GPa, and the tested hardness was lower than the simulated one. This difference was considered to be a consequence of the large number of valence electrons and the mixed ionic/metallic bonding. The TG-DSC measurements enabled us to outline the reaction sequence and latent heat during the evolution of Mn_2_N_0.86_ to Mn_4_N under N_2_ gas protection, which provides a route for the preparation of Mn_4_N. The melting point of Mn_2_N_0.86_ is approximately 650 K, but when heated without the protection of N_2_, it would be oxidised to MnO at temperatures above 500 °C. The simulation suggested that Mn_2_N_0.86_ is antiferromagnetic; however, we determined it to be a combination of chiefly antiferromagnetism and to a lesser extent ferromagnetism owing to N vacancies. This study indicates that by controlling the air pressure, air concentration, and temperature, one could have ideal MnO-Mn_x_N_y_ catalysis and prepare MnN_4_. The magnetic properties of a transition metal nitride could be affected by a very low percentage of N vacancies, which could be utilised as sensors.

## Figures and Tables

**Table 1:** Crystal structure information.

Formula weight	60.96
Crystal system	Hexagonal
Space-group	*P*6_3_22
Cell parameters	a = 4.8641(3) Å c = 4.5269(2) Å
Cell ratio	a/b = 1.0000 b/c = 1.0745
Cell volume	92.76(1) Å^3^
Particle size	139.7 nm
Strain broadening	1.745 × 10^−5^

**Table 2:** The mechanic parameters of Mn_2_N_0.86_.

Phase	C_11_	C_12_	C_13_	C_33_	C_44_	C_66_
Mn_2_N_0.86_	265.711	428.144	191.555	429.358	97.692	189.637
	Bulk Modulus *B* (GPa)	Young’s Modulus *E* (GPa)	Shear Modulus *G* (GPa)	Poisson’s Ratio *V*	Pugh’s Ratio (*B*/*G*)	Vickers Hardness (GPa)
Mn_2_N_0.86_	192.07	285.82	114.15	0.25	1.68	14.26

## Data Availability

The data that support the findings of this study are available from the corresponding author upon reasonable request.
